# CD36-mediated uptake of myelin debris by macrophages and microglia reduces neuroinflammation

**DOI:** 10.1186/s12974-020-01899-x

**Published:** 2020-07-27

**Authors:** Elien Grajchen, Elien Wouters, Britt van de Haterd, Mansour Haidar, Kévin Hardonnière, Tess Dierckx, Jana Van Broeckhoven, Celine Erens, Sven Hendrix, Saadia Kerdine-Römer, Jerome J. A. Hendriks, Jeroen F. J. Bogie

**Affiliations:** 1 Department of Immunology and Infection, Biomedical Research Institute, Hasselt University, Diepenbeek, Belgium; 2 Inflammation, Microbiome and Immunosurveillance, INSERM UMR99, Université Paris-Saclay, Châtenay-Malabry, France

**Keywords:** Macrophages, Microglia, Myelin, CD36, Neuroinflammation, Fatty acid, Multiple sclerosis

## Abstract

**Background:**

The presence of foamy macrophages and microglia containing intracellular myelin remnants is a pathological hallmark of neurodegenerative disorders such as multiple sclerosis (MS). Despite the importance of myelin internalization in affecting both central nervous system repair and neuroinflammation, the receptors involved in myelin clearance and their impact on the phagocyte phenotype and lesion progression remain to be clarified.

**Methods:**

Flow cytometry, quantitative PCR, and immunohistochemistry were used to define the mRNA and protein abundance of CD36 in myelin-containing phagocytes. The impact of CD36 and nuclear factor erythroid 2–related factor 2 (NRF2) on the phagocytic and inflammatory features of macrophages and microglia was assessed using a pharmacological CD36 inhibitor (sulfo-N-succinimidyl oleate) and *Nrf2*^−/−^ bone marrow-derived macrophages. Finally, the experimental autoimmune encephalomyelitis (EAE) model was used to establish the impact of CD36 inhibition on neuroinflammation and myelin phagocytosis in vivo.

**Results:**

Here, we show that the fatty acid translocase CD36 is required for the uptake of myelin debris by macrophages and microglia, and that myelin internalization increased CD36 expression through NRF2. Pharmacological inhibition of CD36 promoted the inflammatory properties of myelin-containing macrophages and microglia in vitro, which was paralleled by a reduced activity of the anti-inflammatory lipid-sensing liver X receptors and peroxisome proliferator-activated receptors. By using the EAE model, we provide evidence that CD36 is essential for myelin debris clearance in vivo. Importantly, CD36 inhibition markedly increased the neuroinflammatory burden and disease severity in the EAE model.

**Conclusion:**

Altogether, we show for the first time that CD36 is crucial for clearing myelin debris and suppressing neuroinflammation in demyelinating disorders such as MS.

## Introduction

The internalization of myelin by phagocytes and the subsequent formation of foamy phagocytes is a pathological hallmark of neurodegenerative disorders [1, 2]. In multiple sclerosis (MS), the presence of foamy phagocytes is even used as an index of MS lesion activity [3]. While myelin internalization was initially regarded to underlie demyelination in neurodegenerative disorders, increasing evidence indicates that clearance of myelin debris by phagocytes is also necessary for central nervous system (CNS) repair [4, 5]. Aside from directly affecting demyelination and remyelination, ample evidence indicates that myelin internalization controls the functional properties of phagocytes as well. For instance, we and others showed that uptake of myelin skews phagocytes towards an immunosuppressive and neurotrophic phenotype [6–8]. This protective phenotype is closely associated with the activation of lipid-responsive signaling pathways, such as the liver X receptor (LXR) and peroxisome proliferator-activated receptor (PPAR) signaling pathways [9–11]. More recent findings indicate that sustained uptake and intracellular accumulation of myelin-derived cholesterol impairs the metabolic and protective features of foamy phagocytes, and instead skews these cells towards an inflammatory, disease-promoting phenotype [12, 13]. Collectively, these findings indicate that myelin internalization is an essential process in the regulation of phagocyte function and disease pathology in demyelinating disorders.

To date, numerous receptors are reported to drive the internalization of myelin. Early studies defined that Fc, complement, and scavenger receptors control myelin phagocytosis [1]. More recent studies indicate that mer tyrosine kinase, low-density lipoprotein receptor-related protein 1, and collectin placenta 1 (CLP1) are also functional regulators of myelin uptake by macrophages and microglia [14–16]. Of interest, many of the abovementioned receptors also regulate the binding and uptake of modified LDL [17–19], Aβ peptides [20–23], and apoptotic cells [24–26]. However, despite being a key receptor in the internalization of these ligands, it remains unclear whether the scavenger receptor CD36 is involved in the phagocytosis of myelin. CD36 is a member of the class B scavenger receptor family and is expressed on various cell types, including monocytes, macrophages, endothelial cells, and adipocytes [27]. *Cd36* deficiency in mice and humans decreases fatty acid uptake in muscle and adipose tissues [28, 29], providing evidence that CD36 acts as a fatty acid translocase. In atherosclerosis, *Cd36* deficiency in macrophages protects mice against lesion development [30–32], consistent with the detrimental role of oxLDL uptake and the formation of foamy macrophages in atherosclerotic lesion progression. Noteworthy, CD36 internalizes oxLDL by a mechanism dependent on the binding of fatty acids [33], suggesting that the presence of fatty acids is important for recognition by CD36. On a transcriptional level, PPARγ and nuclear factor erythroid 2–related factor 2 (NRF2) are amongst the transcription factors that regulate CD36 abundance [34, 35]. Altogether, these studies indicate that CD36 is a phagocytic receptor that plays a vital role in the uptake of fatty acid-containing substrates.

Given the abundance of fatty acids in myelin, we defined in this study if CD36 controls the uptake of myelin by macrophages and microglia. We show that CD36 protein levels are significantly elevated in myelin-containing phagocytes in vitro and in vivo. CD36 protein level was primarily regulated through the NRF2 signaling pathway. Pharmacological inhibition of CD36 reduced the uptake of myelin debris and promoted neuroinflammation in vitro and in vivo. Our findings highlight the importance of functional CD36 in clearing myelin debris and suppressing neuroinflammation in demyelinating CNS disorders such as MS.

## Materials and methods

### Antibodies and chemical reagents

The following antibodies were used for flow cytometry and immunofluorescent/immunohistochemical stainings: anti-CD36 (1:100, cat. #ab80080, Abcam), anti-IBA1 (1:500, cat. #019-19741, Fujifilm), anti-CD68 (1:100, cat. #14-0688, Invitrogen), anti-dMBP (1:2000, cat. #ab5864, Sigma-Aldrich), Alexa Fluor 700-labeled anti-CD45 (1:200, cat. #103128, BioLegend), Zombie NIR (1:1000, cat. #423106, BioLegend), anti-NOS2 (1:100, cat. #ab15323, Abcam), and anti-F4/80 (1:100, cat. #MCA497G, Bio-Rad). Appropriate secondary antibodies were purchased from Invitrogen. BODIPY (493/503) was used to fluorescently label lipid droplets (2 μM, cat. #D3922, Invitrogen). 7-Aminoactinomycin D (7AAD, 0.5 μg/ml, Thermo Fisher Scientific) was used to assess cellular viability. Sulfo-N-succinimidyl oleate (SSO, 100 μM, cat. #11211, Cayman Chemical) and GW9662 (10 μM, cat. #M6191, Sigma-Aldrich) were used to inhibit CD36 and PPARγ, respectively. Lipopolysaccharide (LPS, 100 ng/ml, Sigma-Aldrich) was used to stimulate cells for inflammatory phenotyping.

### Mice

Female wild-type (wt) C57BL/6 J mice were purchased from Envigo. *Nrf2*^−/−^ mice were provided by the RIKEN BRC according to an MTA to Prof S. Kerdine-Römer [35, 36]. Mice were housed in a pathogen-free facility and handled in accordance with the principles and procedures outlined in Council Directive 86/609/EEC. Genotyping was performed by PCR using genomic DNA that was isolated from tail snips as described previously [35, 36]. Mice were maintained on a 12 h light/dark cycle with free access to water and a standard chow diet (Ssniff). All studies were conducted in accordance with the institutional guidelines and approved by the Ethical Committee for Animal Experiments of Hasselt University.

### Experimental autoimmune encephalomyelitis (EAE) model

Twelve-week-old female wt C57BL/6 mice were immunized subcutaneously with 200 μg of recombinant human myelin oligodendrocyte glycoprotein MOG_35–55,_ emulsified in 100 μl complete Freund’s adjuvant supplemented with 4 mg/ml of Mycobacterium tuberculosis (H37RA strain) according to manufacturer’s guidelines (Hooke Laboratories). Within 2 h and after 22–26 h, mice were intraperitoneally injected with 25 ng pertussis toxin. Immunized mice were weighed and scored daily following a five-point standardized rating of clinical symptoms, 0, no signs; 0.5, distal tail paralysis; 1, loss of tail tonus; 2, flaccid tail; 3, hind limb paresis; 4, hind limb paralysis; and 5, death. Starting from day 9 post-immunization or when a disease score of 0.5 or higher was obtained, animals were injected intraperitoneally with SSO (30 mg/kg) or vehicle on a daily basis. Mice were sacrificed at day 22 post-immunization or at 11 days post-disease onset.

### Cell isolation and culture

Bone marrow-derived macrophages (BMDMs) were obtained as described previously [14]. Briefly, femoral and tibial bone marrow cell suspensions from 12-week-old wt and *Nrf2*^−/−^ C57Bl/6 mice were cultured in 10 cm plates at a concentration of 10 × 10^6^ cells/plate and differentiated in RPMI 1640 medium (Lonza) supplemented with 10% fetal calf serum (FCS, Gibco), 50 U/ml penicillin (Invitrogen), 50 U/ml streptomycin (Invitrogen), and 15% L929-conditioned medium (LCM). After differentiation, cells were cultured (0.5 × 10^6^ cells/ml) in RPMI 1640 supplemented with 10% FCS, 50 U/ml penicillin, 50 U/ml streptomycin, and 5% LCM. Primary microglia cell cultures were prepared from brains of postnatal P1-3 C57BL/6 pups. Carefully, the brain stem, choroid plexus, and meninges were removed before brains were enzymatically digested for 15 min with 1x trypsin (Gibco) at 37 °C. The tissue was mechanically dissociated in DMEM high glucose medium (Sigma) supplemented with 10% FCS, 50 U/ml penicillin, and 50 U/ml streptomycin (DMEM complete), after which the cell suspension was seeded out in T75 culture flasks. Two to three days later, a complete medium change was performed. When 80% confluency was reached, cells were cultured in DMEM complete supplemented with 30% LCM. Microglia were obtained 6–7 days later after shake-off (230 rpm, 3 h, 37 °C). Cells were cultured (0.4 × 10^6^ cells/ml) in DMEM complete supplemented with 15% LCM. For the isolation of CNS-derived leukocytes from EAE animals, the brain and spinal cord were cut into pieces and further homogenized in HBSS supplemented with DNAse. Single-cell suspensions were obtained by pushing the homogenate through a 70µm cell strainer. A discontinuous Percoll gradient (90%, 60%, and 40%) was used to separate cells from myelin and debris. The cells were gently collected from the 40–60% interface and washed for further flow cytometric analysis.

### Myelin isolation and phagocytosis

Myelin was purified from postmortem mouse brain tissue by means of density gradient centrifugation, as described previously [37]. Myelin protein concentration was determined using the BCA protein assay kit (Thermo Fisher), according to manufacturer’s guidelines. By means of the Chromogenic Limulus Amebocyte Lysate assay kit (Genscript Incorporation), endotoxin content of isolated myelin was determined to be negligible. Cells were treated with 100 μg/ml myelin. To evaluate the ability and extent of myelin phagocytosis, myelin was fluorescently labeled with 1,1′-dioctadecyl-3,3,3′,3′-tetramethylindocarbocyanine perchlorate (DiI, Sigma)*.* Cells were exposed to 100 μg/ml DiI-labeled myelin for 1.5 h and analyzed for fluorescence intensity by using the FACSCalibur (BD Biosciences).

### Flow cytometry

Single-cell suspensions were blocked with 10% serum and stained with relevant primary antibodies, followed by incubation with the appropriate secondary antibody. To assess lipid load in CNS-derived leukocytes, single-cell suspensions were incubated with Zombie NIR for 15 min, followed by 15 min incubation with Alexa Fluor 700-labeled anti-CD45. Finally, cells were stained for intracellular lipid load by 15 min incubation with BODIPY (493/503) at 37 °C. To analyze cellular viability, cells were incubated with 7AAD for 10 min. The FACSCalibur or BD LSRFortessa was used to quantify cellular fluorescence.

### Quantitative PCR

Total RNA from cultures was prepared using the RNeasy mini kit (Qiagen), according to manufacturer’s instructions. RNA quality was assessed with a NanoDrop spectrophotometer (Isogen Life Science). RNA was converted to cDNA using the reverse transcription system (Quanta Biosciences) and quantitative PCR was subsequently performed on a StepOnePlus detection system (Applied Biosystems). Relative quantification of gene expression was accomplished using the comparative C_t_ method. Data were normalized to the most stable reference genes, TATA binding protein (*Tbp*), and cyclin A (*Cyca*) [38]. Primer sequences are available on request.

### Nitrite formation

To measure the release of NO in supernatants from cell cultures, culture medium was added to an equal volume of 0.05% N-(1-Naphthyl)ethylenediamine dihydrochloride, 0.5% sulfanilamide, and 2.5% phosphatidic acid solution. Absorbance was measured at 540 nm.

### Immunofluorescence

Murine BMDMs and microglia were cultured on glass cover slides and fixed in 4% paraformaldehyde for 20 min. Spinal cord tissue of EAE mice was isolated, snap-frozen, and sectioned with a Leica CM1900UV cryostat (Leica Microsystems) to obtain 8 μm slices. Frozen human brain material from active MS lesions was obtained from the Netherlands Brain Bank (NBB, Amsterdam, Netherlands). Cryosections were fixed in acetone for 10 min and in 70% ethanol for 5 min. Immunostaining and analysis of fixed cells and cryosections were performed as described previously [14]. Analysis was carried out using a Nikon eclipse 80i microscope and ImageJ software. Quantification of mean fluorescence intensity (MFI) or percentage positivity was carried out on original images without enhancement. Images shown in figures are digitally enhanced.

### Immunohistochemistry

For 3, 3′ diaminobenzidine (DAB) staining, slides were incubated with rat anti-CD36. After washing, HRP-conjugated goat anti-rat (Dako) was added. Subsequently, DAB substrate (Dako) was used to stain slides. Intracellular myelin degradation products were visualized with Oil Red O (ORO), which stains neutral lipids. For this purpose, cells and tissue were stained with 0.3% ORO (Sigma) for 10 min. Cells and cryosections were counterstained with hematoxylin (Merck). Images were acquired using a Leica DM 2000 LED microscope and a Zeiss LSM880 confocal microscope, and quantitative analysis was carried out using the ImageJ software.

### Statistical analysis

Data were statistically analyzed using GraphPad Prism for windows and are reported as mean ± s.e.m. D’Agostino and Pearson omnibus normality test was used to test normal distribution. An analysis of variances (ANOVA) or two-tailed unpaired student *t* test (with Welch’s correction if necessary) was used for normally distributed data sets. The Kruskal-Wallis or Mann-Whitney analysis was used for data sets which did not pass normality. *p* values < 0.05 were considered to indicate a significant difference (*p < 0.05, **p < 0.01, and ***p < 0.001).

## Results

### Myelin uptake increases CD36 abundance in phagocytes in an NRF2-dependent manner

**Fig. 1:**
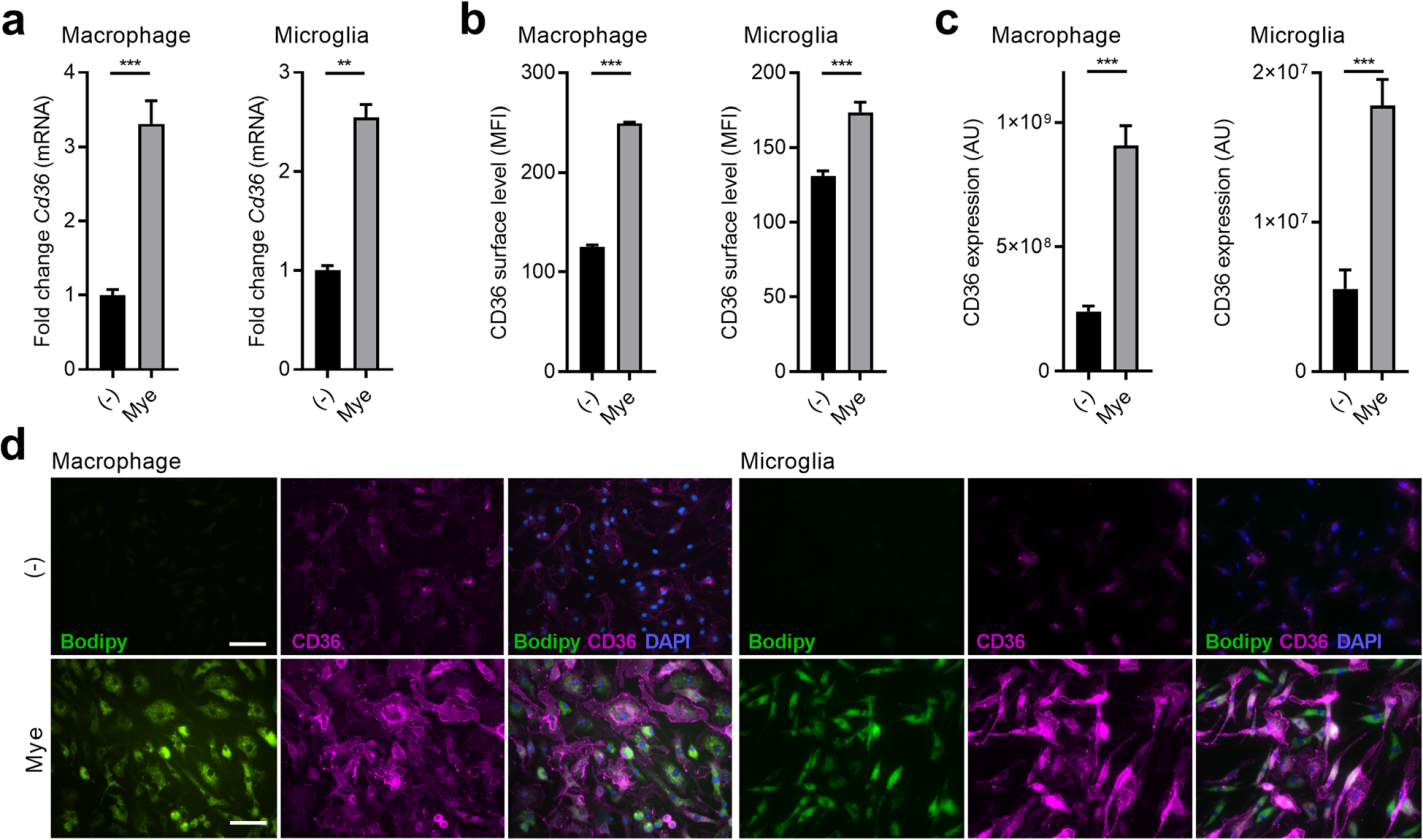
Myelin internalization increases CD36 abundance in phagocytes in vitro. **a** mRNA expression of *Cd36* in bone marrow-derived macrophages (BMDMs, *n* = 10 wells) and microglia (*n* = 6 wells) treated with myelin or left untreated for 24 h. **b** Flow cytometric analysis of surface CD36 abundance on BMDMs (*n* = 5 wells) and microglia (*n* = 8 wells) treated with myelin or left untreated for 24 h. **c**, **d** Quantification (fluorescence intensity) and representative images of CD36 abundance in BMDMs (*n* = 80 cells) and microglia (*n* = 80 cells) treated with myelin or left untreated for 24 h. Scale bar, 40 μm. MFI, mean fluorescence intensity; AU, arbitrary units. Data are represented as mean ± s.e.m. **p < 0.01 and ***p < 0.001

**Fig. 2:**
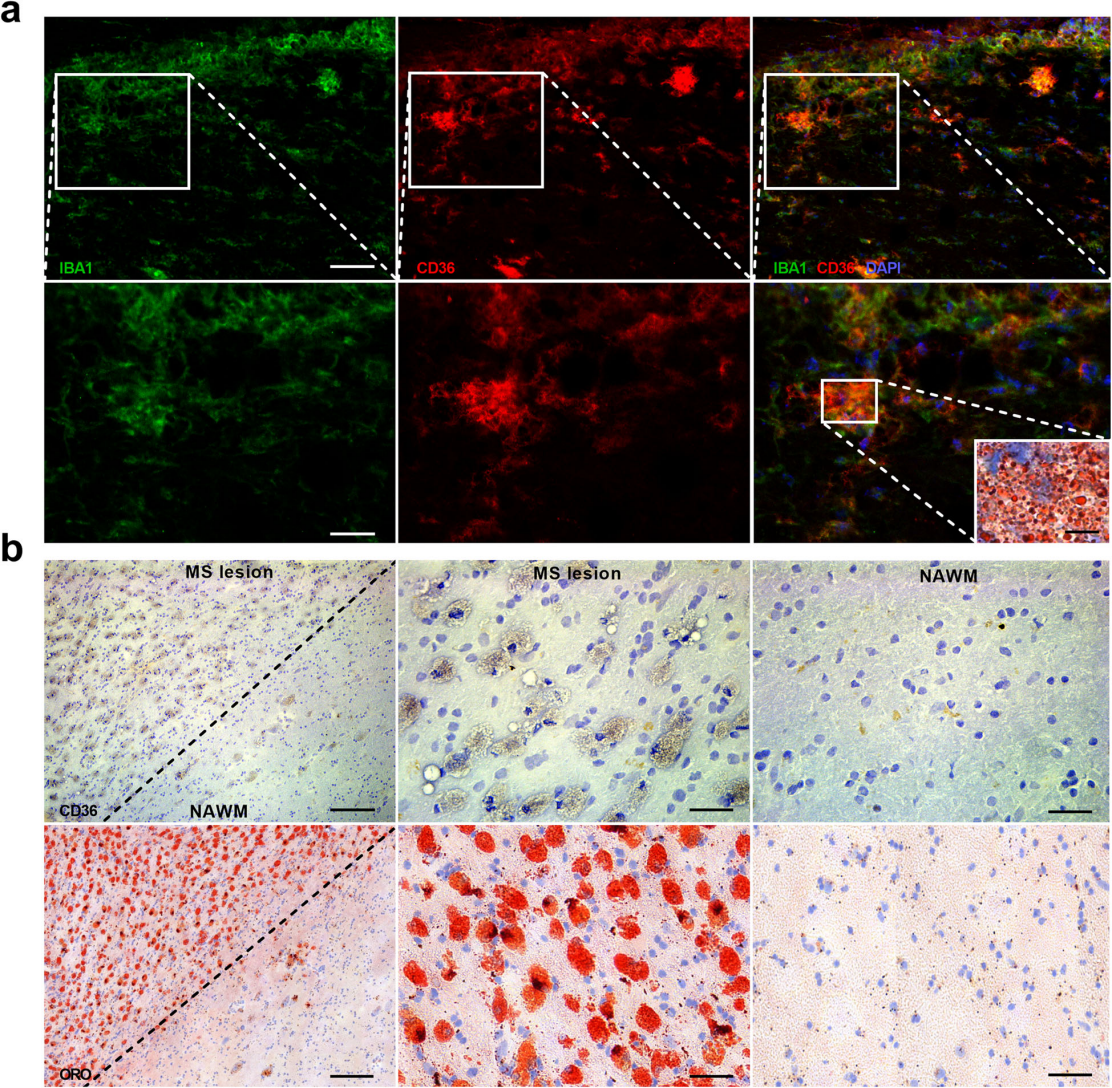
Myelin internalization increases CD36 abundance in phagocytes in vivo*.***a** Representative immunofluorescent images of a spinal cord lesion of an EAE animal (28 days post-immunization) stained with IBA1/CD36 or Oil Red O (ORO). Scale bars, 50 μm (top images); 25 μm (bottom images); 10 μm (ORO inlet). **b** Representative images of an active MS lesion stained with CD36 (top images) and ORO (bottom images). Scale bars, 100 μm (left images); 50 μm (middle and right images)

The internalization of oxLDL, Aβ peptides, and apoptotic cells is well-known to increase CD36 expression in phagocytes [17–26]. To define whether myelin internalization also increases CD36 expression in macrophages and microglia, quantitative PCR, flow cytometry, and immunostaining were used. Our data show that myelin uptake induced mRNA expression of *Cd36* in murine bone marrow-derived macrophages (BMDMs) and microglia (Fig. 1a). In line with these findings, myelin-treated BMDMs and microglia had elevated protein levels of CD36 (Fig. 1b–d). Also, within demyelinating lesions in the experimental autoimmune encephalomyelitis (EAE) mouse model, foamy IBA1-expressing phagocytes highly expressed CD36 (Fig. 2a). To obtain human validation, active MS lesions that are characterized by the abundant presence of myelin-containing phagocytes were stained for CD36 (Fig. 2b). Immunohistochemical staining demonstrated that CD36 was primarily expressed in highly granular cells resembling myelin-containing phagocytes (Fig. 2b).

**Fig. 3:**
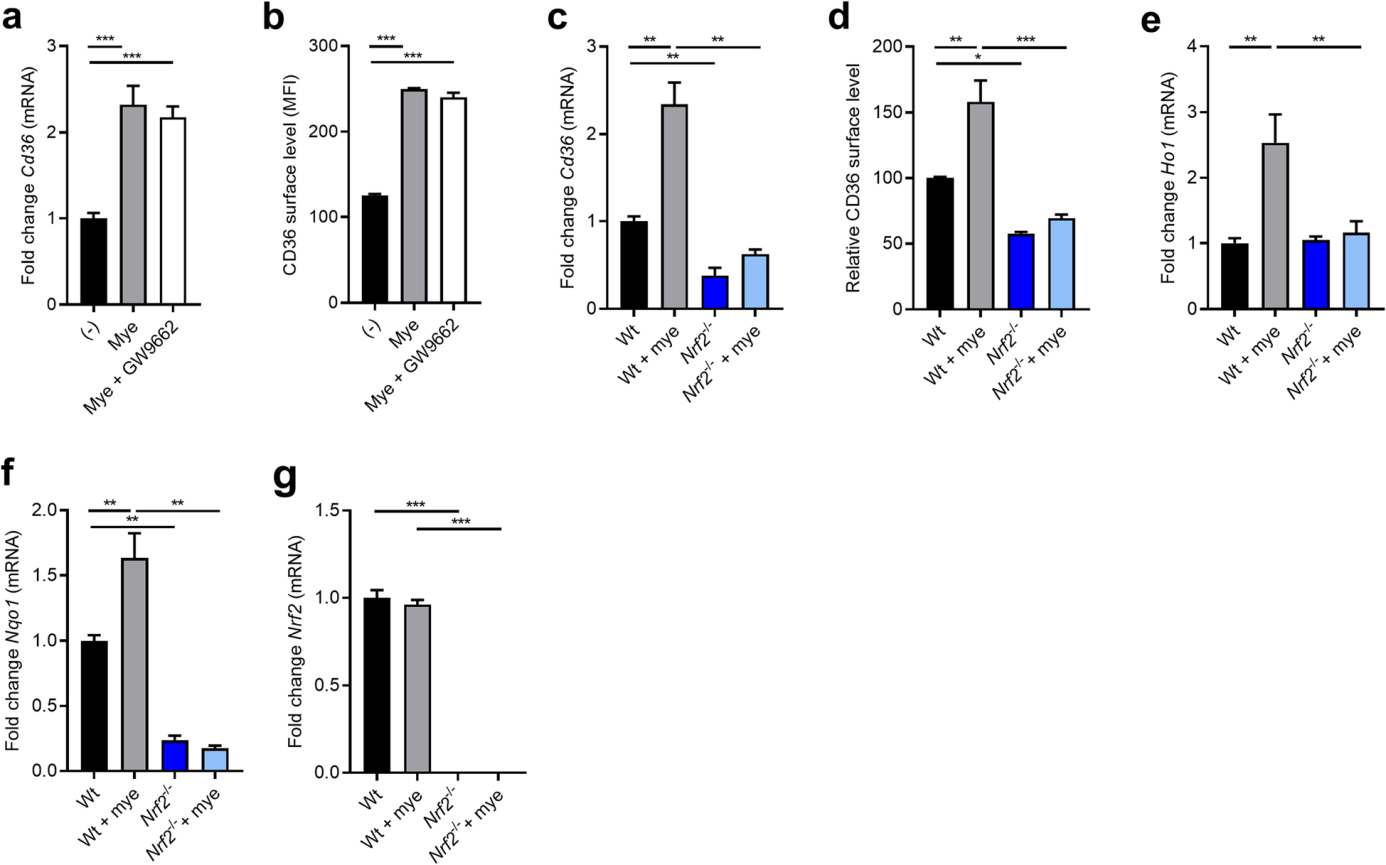
Myelin uptake increases CD36 expression in an NRF2-dependent manner. **a**, **b** mRNA expression (*n* = 9 wells) and surface protein level (*n* = 4 wells) of CD36 in bone marrow-derived macrophages (BMDMs) treated with vehicle, myelin, and a PPARγ antagonist (GW9662, 10 μM) for 24 h (*n* = 9 wells). **c**, **d** mRNA expression (*n* = 6 wells) and relative surface protein level (*n* = 4 wells) of CD36 in wild-type (wt) and *Nrf2*-deficient (*Nrf2*^−/−^) BMDMs treated with myelin or left untreated for 24 h. **e**–**g** mRNA expression (*n* = 6 wells) of *Ho1*, *Nqo1*, and *Nrf2* in wt and *Nrf2*^−/−^ BMDMs treated with myelin or left untreated for 24 h. MFI, mean fluorescence intensity. Data are represented as mean ± s.e.m. *p < 0.05, **p < 0.01, and ***p < 0.001

Previous studies defined that PPARγ and NRF2 control CD36 expression levels [34, 35]*.* By using an antagonist of PPARγ (GW9662), we found that myelin did not increase CD36 mRNA expression and protein abundance through PPARγ (Fig. 3a and b). In contrast, a marked decrease in CD36 mRNA and protein levels was observed in myelin-treated* Nrf2*^−/−^ BMDMs as compared to wild-type (wt) BMDMs (Fig. 3c and d). In line with active NRF2 signaling, myelin-treated BMDMs showed an elevated mRNA expression of heme oxygenase 1 (*Ho1*) and NAD(P)H:quinone acceptor oxidoreductase 1 (*Nqo1*) (Fig. 3e and f), which was not observed in *Nrf2*^−/−^ BMDMs. Myelin internalization did not change the expression of *Nrf2* (Fig. 3g), indicating that intracellular myelin accumulation promotes the activation but not expression of NRF2. Collectively, these findings show that myelin increases CD36 expression on gene and protein level in an NRF2-dependent manner.

### The NRF2-CD36 signaling axis controls the uptake of myelin by phagocytes

**Fig. 4:**
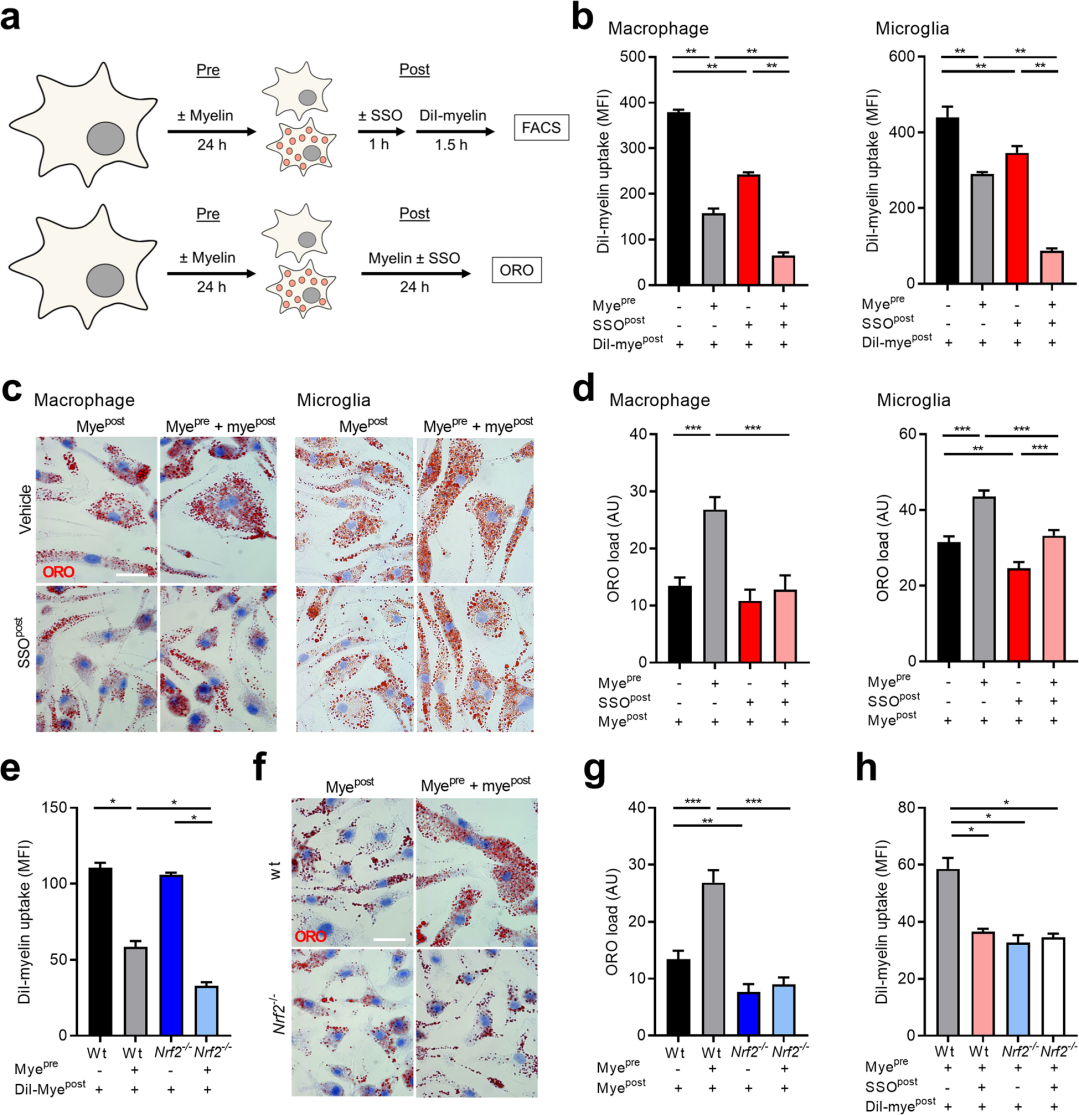
The NRF2-CD36 signaling axis controls myelin internalization by phagocytes. **a** Experimental setup of phagocytosis (top) and Oil Red O (ORO, bottom) experiments. **b** Internalization of DiI-labeled myelin by control and myelin pre-treated bone marrow-derived macrophages (BMDMs, *n* = 6 wells) and microglia (*n* = 4 wells). The CD36 inhibitor sulfo-N-succinimidyl oleate (SSO, 100 μM) was used to block CD36 activity. The internalization of DiI-labeled myelin was defined via flow cytometry and data are depicted as mean fluorescence intensity (MFI). **c**, **d** Representative images and quantification of ORO staining of control and myelin pre-treated BMDMs (*n* = 20 cells) and microglia (*n* = 50 cells) exposed to SSO (100 μM) or vehicle and myelin. ORO load is defined as the percentage of cellular area that is ORO^+^. Scale bar, 20 μm. **e** Internalization of DiI-labeled myelin by wild-type (wt) and *Nrf2*^−/−^ control and myelin pre-treated BMDMs (*n* = 4 wells). **f**, **g** Representative images and quantification of ORO staining of control and myelin pre-treated wt and *Nrf2*^−/−^ BMDMs exposed to myelin (*n* = 20 cells). Scale bar, 20 μm. **h** Internalization of DiI-labeled myelin by myelin pre-treated wt and *Nrf2*^−/−^ BMDMs, which were exposed to SSO (100 μM) or vehicle (*n* = 4 wells). AU, arbitrary units. Data are represented as mean ± s.e.m. *p < 0.05 and **p < 0.01

CD36 is a phagocytic receptor that mediates the uptake of fatty acid-containing ligands [33]. Given the abundance of fatty acids in myelin, we next determined if CD36 controls the uptake of myelin by macrophages and microglia as well. By using sulfo-N-succinimidyl oleate (SSO), a pharmacological CD36 inhibitor (experimental design Fig. 4a), we show that CD36 mediates the uptake of myelin by control macrophages and those pre-pretreated with myelin (Fig. 4b), without affecting cell viability (Supplemental Fig. 1). Macrophages pre-treated with myelin showed a reduced phagocytic capacity as compared to control cells, suggesting that myelin internalization activates inhibitory processes that limit the excessive uptake of myelin (Fig. 4b). Similar findings were obtained using primary mouse microglia (Fig. 4b and Supplemental Fig. 1). Notably, CD36 inhibition decreased the phagocytic capacity of macrophages and microglia pre-treated with myelin more prominently than control cells (macrophages, 40% (control) vs 60% (myelin pre-treated) reduction; microglia, 34% (control) vs 75% (myelin pre-treated) reduction). The latter finding is consistent with the elevated CD36 protein level in these phagocytes following myelin exposure. In line with the reduced phagocytic capacity upon CD36 inhibition, abrogation of CD36 signaling markedly reduced the intracellular lipid load in macrophages upon exposure to myelin (Fig. 4c and d, experimental design Fig. 4a). In summary, these findings indicate that CD36 is involved in the clearance of myelin debris by macrophages and microglia.

Given the importance of NRF2 in controlling CD36 abundance, we next determined if NRF2 underlies the role of CD36 in the clearance of myelin debris. We provide evidence that *Nrf2* deficiency reduces myelin internalization by BMDMs to a similar extent as CD36 inhibition (Fig. 4e). In concordance, neutral lipid levels were significantly decreased in myelin-treated *Nrf2*^−/−^ BMDMs compared to wt BMDMs (Fig. 4f and g). Importantly, pharmacological inhibition of CD36 was unable to further decrease the clearance of myelin debris by *Nrf2*^−/−^ macrophages (Fig. 4h). These findings demonstrate that CD36 controls myelin uptake by macrophages in an NRF2-dependent manner.

### CD36-mediated myelin uptake skews macrophages and microglia towards a less-inflammatory phenotype

**Fig. 5:**
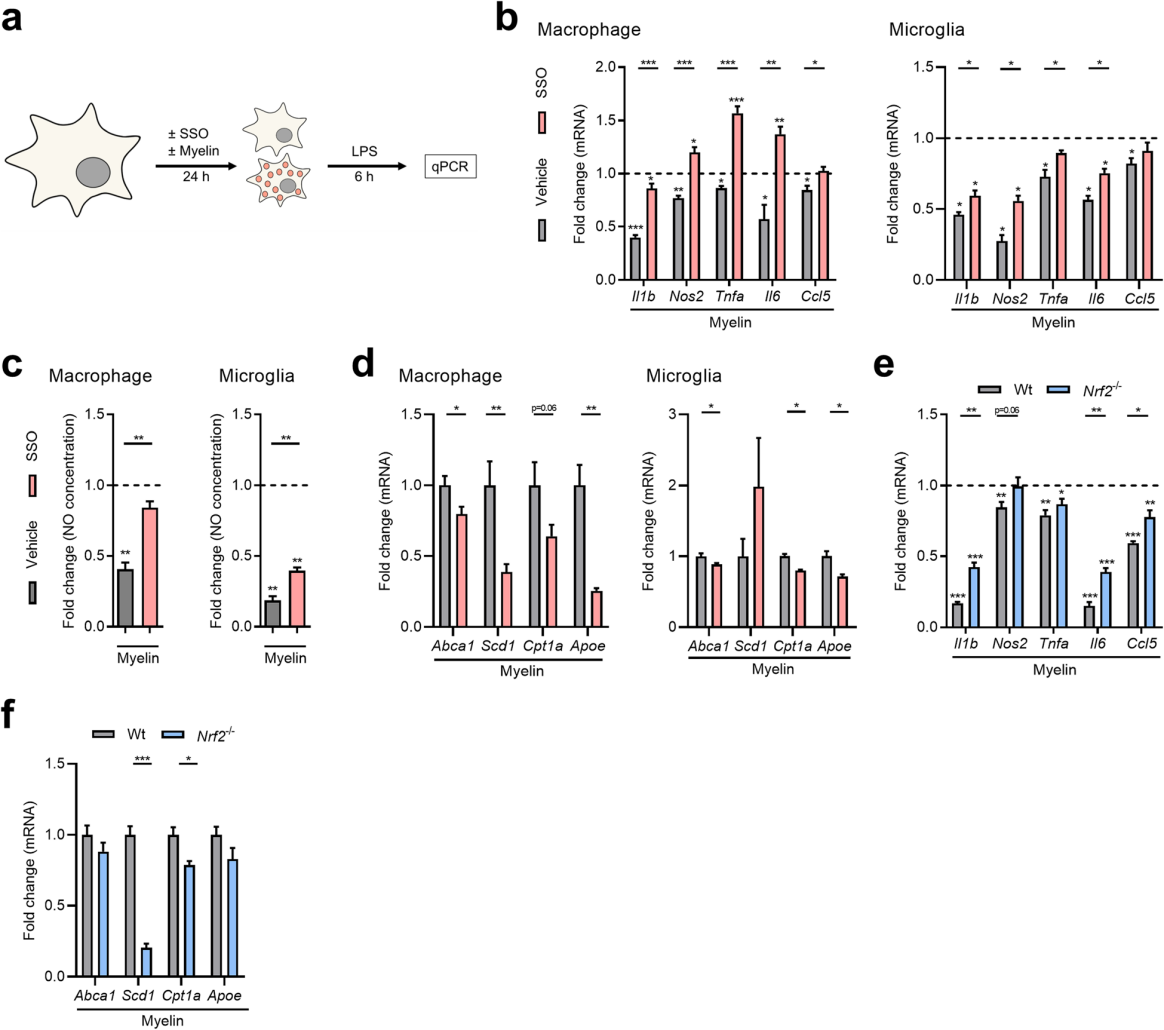
CD36 inhibition and *Nrf2* deficiency counters the protective phenotype of myelin-containing phagocytes. **a** Experimental setup used to define the impact of CD36 inhibition on the inflammatory and metabolic phenotype of myelin-containing bone marrow-derived macrophages (BMDMs) and microglia. **b** mRNA expression of *Il1b*, *Nos2*, *Tnfa*, *Il6*, and *Ccl5 *in LPS-stimulated BMDMs (*n* = 6 wells) and microglia (*n* = 4 wells) treated with vehicle, myelin, and the CD36 inhibitor sulfo-N-succinimidyl oleate (SSO, 100 μM) for 24 h. Dotted line represents control cells stimulated with LPS. **c** NO concentration in culture supernatants of LPS-stimulated BMDMs (*n* = 6 wells) and microglia (*n* = 5 wells) treated with vehicle, myelin, and SSO (100 μM). **d** mRNA expression of *Abca1*, *Scd1*, *Cpt1a*, and *Apoe* in LPS-stimulated BMDMs (*n* = 8 wells) and microglia (*n* = 4 wells) treated with vehicle, myelin, and the CD36 inhibitor for 24 h. **e** mRNA expression of *Il1b*, *Nos2*, *Tnfa*, *Il6*, and *Ccl5* in LPS-stimulated BMDMs isolated from *Nrf2*^−/−^ mice and wild-type (wt) littermates treated with vehicle and myelin (*n* = 5 wells). Dotted line represents control cells stimulated with LPS. **f** mRNA expression of *Abca1*, *Scd1*, *Cpt1a*, and *Apoe* in LPS-stimulated wt and *Nrf2*^−/−^ BMDMs treated with myelin (*n* = 5 wells). Data are represented as mean ± s.e.m. *p < 0.05, **p < 0.01, and ***p < 0.001

Ample evidence indicates that myelin uptake skews phagocytes towards a less-inflammatory phenotype [6–8]*.* Here, we confirm that intracellular myelin accumulation reduces the inflammatory status of LPS-stimulated macrophages and microglia (experimental design Fig. 5a), evidenced by a reduced mRNA expression of *Il1b*, *Nos2*, *Tnfa*, *Il6*, and *Ccl5* (Fig. 5b), and a decreased presence of NO in the culture medium (Fig. 5c). Importantly, we show that CD36 inhibition counters the anti-inflammatory impact of myelin on the phenotype of LPS-stimulated as well as unstimulated phagocytes (Fig. 5b and c, Supplemental Fig. 2a). SSO did not impact the inflammatory phenotype of empty macrophages (Supplemental Fig. 2b). Previously, we showed that myelin-mediated activation of LXRs and PPARs underlies the anti-inflammatory impact of myelin on the phenotype of macrophages [9–11]*.* Consistent with these findings, CD36 inhibition reduced the expression of the LXR- and PPAR-responsive genes *Abca1*, *Scd1*, *Cpt1a*, and *Apoe* in myelin-treated macrophages (Fig. 5d). Similar findings were observed when using microglia, with the exception of *Scd1* expression (Fig. 5d). Interestingly, absence of *Nrf2* induced a similar inflammatory and metabolic phenotype in myelin-phagocytosing macrophages (Fig. 5e and f), pointing towards a key role of the NRF2-CD36 signaling axis in driving the phenotype of foamy phagocytes. Collectively, these findings indicate that CD36 inhibition skews myelin-containing macrophages towards an inflammatory phenotype, potentially by reducing the activation of LXRs and PPARs.

### CD36 inhibition promotes neuroinflammation in vivo

**Fig. 6:**
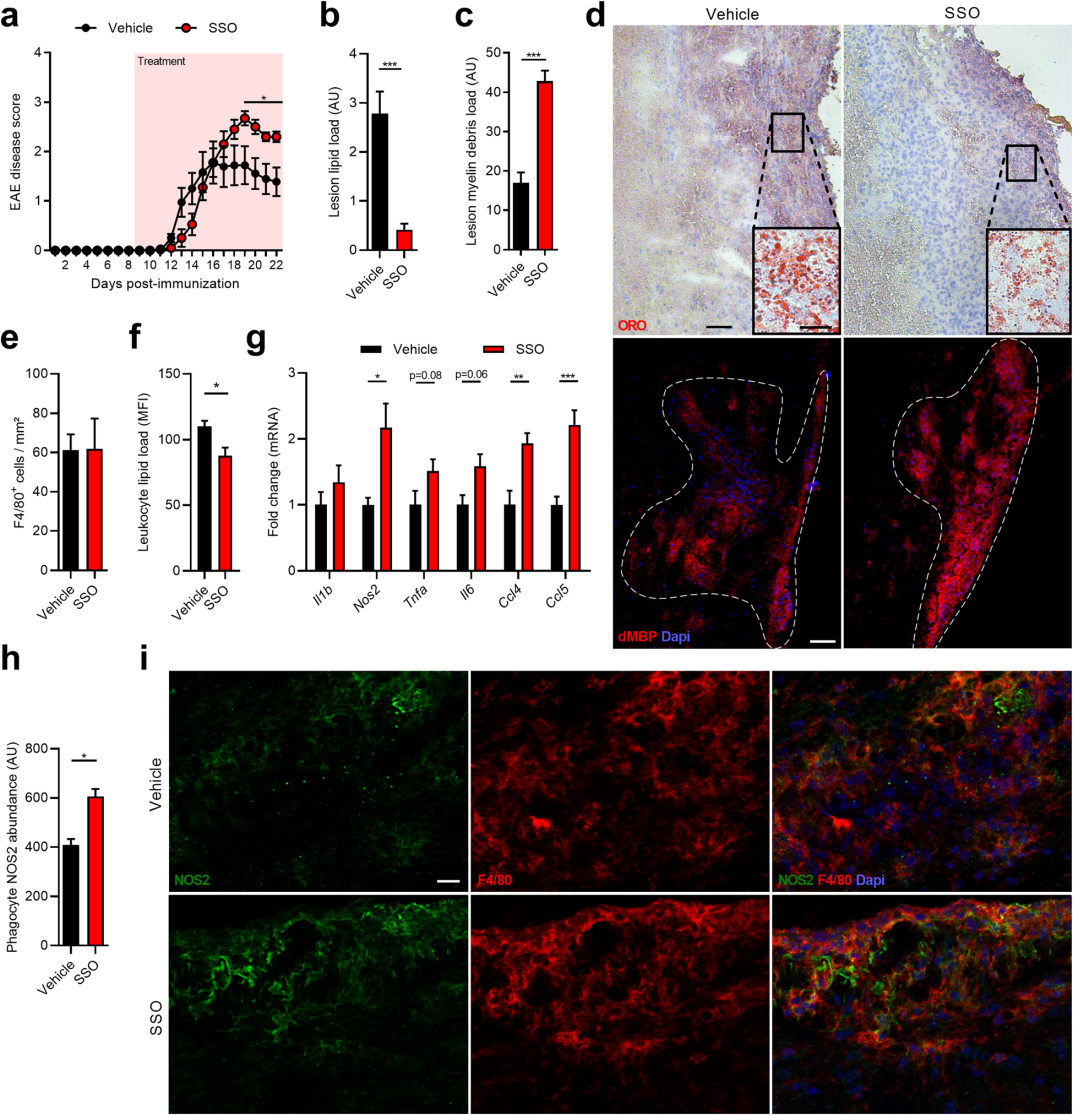
CD36 inhibition increases neuroinflammation and disease severity in the experimental autoimmune encephalomyelitis animal model. **a** Disease score of 12-week-old wild-type (wt) mice in which experimental autoimmune encephalomyelitis (EAE) was induced. Starting 9 days post-immunization, animals were injected intraperitoneally with vehicle (*n* = 9 animals) or the CD36 inhibitor sulfo-N-succinimidyl oleate (SSO, 30 mg/kg, *n* = 10 animals) on a daily basis. **b**–**d** Quantification and representative images of an Oil Red O (ORO) staining (**b**, **d**, *n* = 6 lesions) and degenerated myelin (dMBP) staining (**c**, **d**, *n* = 5 animals) of spinal cord tissue obtained from EAE animals treated with vehicle or CD36 inhibitor. Dotted line delineates the circumference of the lesion. Lesion lipid and myelin debris load are defined as the percentage of lesion area that is ORO^+^ and dMBP^+^, respectively. Scale bars, 100 μm; 25 μm (inlet images). **e** Quantification of F4/80 staining of spinal cord tissue obtained from EAE animals treated with vehicle or CD36 inhibitor (*n* = 5 animals). **f** Flow cytometric analysis of intracellular lipid load (BODIPY stain) in CD45^+^ leukocytes (*n* = 4 animals). Data are depicted as mean fluorescence intensity (MFI). **g** Quantitative PCR was used to assess mRNA expression of *Il1b*, *Nos2*, *Tnfa*, *Il6*, *Ccl4*, and *Ccl5* spinal cord tissue obtained from EAE animals treated with vehicle or CD36 inhibitor (*n* = 6 animals). **h**, **i** Quantification and representative images of NOS2/F4/80 staining of spinal cord tissue obtained from EAE animals treated with vehicle or CD36 inhibitor (*n* = 5 animals). Phagocyte NOS2 abundance is defined as the NOS2 MFI of F4/80^+^ cells. Scale bar, 25 μm. Data are represented as mean ± s.e.m. *p < 0.05, **p < 0.01, and ***p < 0.001

Our findings indicate that CD36 inhibition reduces clearance of myelin debris and skews myelin-treated macrophages towards an inflammatory phenotype. To validate our in vitro findings, we next determined the impact of CD36 inhibition on neuroinflammation using the EAE mouse model. Animals treated with the CD36 inhibitor before EAE onset showed a worsened EAE disease severity in the chronic phase compared to vehicle-treated animals (Fig. 6a). Immunohistochemical analysis of spinal cord lesions of EAE animals treated with the CD36 inhibitor showed a reduced presence of intracellular ORO^+^ lipid droplets and increased immunoreactivity for degenerated myelin (dMBP) (Fig. 6b–d), with no difference in the number of F4/80^+^ phagocytes (Fig. 6e). Flow cytometric analysis demonstrated that CD45^+^ leukocytes in the spinal cord of SSO-treated animals had a reduced intracellular lipid load (Fig. 6f). These findings strongly suggest that CD36 inhibition reduces the phagocytosis of myelin debris in vivo. Furthermore, CD36 inhibition increased the neuroinflammatory burden in the spinal cord of EAE animals, as shown by a significantly elevated expression of *Nos2*, *Ccl4*, and *Ccl5*, along with a trend towards increased mRNA levels of *Tnfa* and *Il6* (Fig. 6g)*.* In line with the elevated *Nos2* mRNA expression, treatment with the CD36 inhibitor increased NOS2 reactivity in F4/80^+^ macrophages and microglia within spinal cord lesions (Fig. 6h and i). To assess the therapeutic efficacy of CD36 inhibition, EAE animals were treated with SSO after disease onset. In contrast to the prophylactic treatment regime, our findings indicate that SSO treatment does not reduce disease EAE disease severity once clinical symptoms have established (Supplemental Fig. 3). In summary, these findings show that CD36 regulates the uptake of myelin debris by phagocytes in vivo. Furthermore, they suggest that CD36-mediated clearance of myelin debris reduces neuroinflammation by driving phagocytes towards a less-inflammatory phenotype, in particular in early disease stages.

## Discussion

Foamy phagocytes containing myelin remnants are abundantly present in active MS lesions. To date, the receptors that are involved in myelin clearance and their impact on the macrophage phenotype and lesion progression remain to be clarified. Here, we show that fatty acid translocase CD36 controls the uptake of myelin debris by phagocytes and subsequently controls its own expression in an NRF2-dependent manner. Pharmacological inhibition of CD36 countered the initial protective phenotype that phagocytes adopt after myelin uptake, resulting in increased EAE disease severity. Exacerbated neuroinflammation was closely associated with a reduced intracellular phagocytic myelin load and increased presence of myelin debris in the spinal cord lesions. Thus, our findings show that CD36 plays a protective role in demyelinating disorders by driving the clearance of myelin debris and suppressing the induction of inflammatory phagocytes.

CD36 is a scavenger receptor involved in the uptake of oxLDL by a mechanism dependent on fatty acid binding [33]. Our findings now indicate that CD36 also mediates the internalization of myelin debris. Given the abundant presence of fatty acids in myelin, it is tempting to speculate that CD36-mediated clearance of myelin depends on fatty acid recognition as well. With respect to the latter, CD36 mediates the uptake of mono- and poly-unsaturated long-chain fatty acids such as oleic and docosahexaenoic acid [39, 40], which are highly present in myelin [41]. In future studies, it would be of interest to define the fatty acid substrates in myelin recognized by CD36, and to what extent the levels of these fatty acids are altered in MS. Moreover, as the phagocytosis experiments carried out in this manuscript were performed using myelin debris, future studies should define whether these fatty acid substrates also underlie internalization of intact myelin or whether they are only accessible for CD36 recognition in damaged myelin.

We show that myelin uptake increases CD36 gene expression and protein levels in macrophages and microglia in vitro and within EAE and MS lesions. Correspondingly, CNS lesions in experimental models for ischemic stroke and spinal cord injury (SCI), both characterized by high numbers of myelin-containing cells, show increased levels of CD36 [42, 43]. Interestingly, these findings indicate a positive feedback loop in which CD36-mediated myelin uptake boosts its own expression and secondary uptake of myelin. A similar positive feedback loop has previously been described for CD36 in oxLDL-loaded macrophages in atherosclerotic lesions [44]. Moreover, we recently found that the scavenger receptor CLP1 mediates the uptake of myelin, which subsequently increased its own expression [45]. Collectively, these findings suggest that myelin internalization increases CD36 abundance, thereby sustaining continuous clearance of myelin debris.

Our findings indicate that NRF2 increases CD36 expression in macrophages and microglia upon myelin debris internalization and underlies the impact of CD36 on the phagocytic capacity and inflammatory phenotype of myelin-phagocytosing macrophages. These results are in line with other studies showing the importance of NRF2 in controlling CD36 expression in innate immune cells [35, 46, 47]. The NRF2 pathway is a major regulator of cytoprotective responses to endogenous and exogenous stresses caused by reactive oxygen species (ROS) [48]. On this note, van der Goes et al. demonstrated that myelin internalization triggers the production of ROS, and that scavenging and blocking of ROS reduce the phagocytosis of myelin by macrophages [49]. This study emphasizes the essential role of the oxidative stress response in myelin phagocytosis and provides a rationale for the increased NRF2 activity observed in our cultures. Surprisingly, even though PPARγ controls CD36 expression and myelin-phagocytosing macrophages show active PPAR signaling [9, 34], the expression of CD36 in myelin-containing macrophages was not affected by a PPARγ antagonist. Accordingly, we previously demonstrated that PPARγ inhibition does not affect the inflammatory phenotype of myelin-containing macrophages [9]. In summary, our findings highlight the importance of NRF2 in controlling CD36 abundance in macrophages upon myelin internalization.

Previously, we and others showed that myelin uptake induces a less-inflammatory, reparative phenotype via the activation of the lipid-sensing nuclear receptors LXRs and PPARs [9, 10]. Here, we report that pharmacological inhibition of CD36 activity prevents the induction of the less-inflammatory phenotype of macrophages in vitro, and in parallel, reduces the expression of LXR- and PPAR-responsive genes in these cells. A reduced uptake of myelin-derived cholesterol and fatty acids and a consequent decreased LXR and PPAR activity might well explain the inflammatory phenotype of macrophages upon CD36 inhibition in vitro.

Ample evidence indicates that macrophages and microglia have both disease-promoting and resolving functions in demyelinating CNS disorders [1]. Given the inhibitory role of myelin debris on oligodendrocyte maturation, the clearance of damaged myelin debris by phagocytes is considered to be essential for CNS repair [5, 50]. Here, we show that CD36 inhibition increases disease severity at the chronic disease stage of EAE when treatment started before disease onset. Aggravated disease severity was associated with a reduced phagocyte myelin load, increased presence of non-cell associated degenerated myelin, and elevated neuroinflammatory burden in the CNS. These findings strongly suggest that CD36 inhibition reduces the clearance of myelin debris by phagocytes and promotes their inflammatory phenotype in vivo. In line with our findings, Zhu et al. showed that *Cd36* deficiency in the SCI mouse model also reduced lesional phagocyte lipid content. However, in this study absence of *Cd36* improved lesion size and locomotor recovery [42]. One possible explanation for this ambiguity is the difference in lesional phagocytic lipid load between these models. In the EAE model, the extent of demyelination is relatively limited compared to the SCI model. On this note, we showed that the intracellular myelin load determines the inflammatory phenotype of macrophages and microglia [13]. It is tempting to speculate that blocking myelin uptake in the SCI model is beneficial because the induction of inflammatory features associated with an elevated intracellular myelin overload is prevented. Notably, our findings further indicate that that CD36 inhibition does not affect EAE disease severity once administrated after disease onset. The extensive amount of demyelination and thus phagocytic myelin uptake in the early, non-symptomatic disease stage might well explain why CD36 inhibition had no effect in this experimental setup [51, 52]. More research is necessary to characterize the underlying mechanism of the beneficial and harmful effects of myelin clearance in different diseases and model systems, and to define the optimal timing of CD36 inhibitor administration.

## Conclusion

Our study provides the first evidence for an essential role of CD36 in the uptake of myelin and in controlling the inflammatory properties of phagocytes in demyelinating disorders. By mediating myelin debris clearance, CD36 induces a protective phenotype in myelin-laden macrophages and microglia, and dampens EAE disease severity. Hence, targeting CD36 holds therapeutic promise for demyelinating disorders such as MS.

## Supplementary information

**Additional file 1:Supplemental Figure S1.** CD36 inhibition does not affect phagocyte viability. Percentage of viable bone marrow-derived macrophages (BMDMs, *n* = 6 wells) and microglia (*n* = 4 wells) after treatment with vehicle or the CD36 inhibitor sulfo-N-succinimidyl oleate (SSO, 100 μM) for 24 h. Data are represented as mean ± s.e.m.

**Additional file 2: Supplemental Figure S2.** CD36 inhibition modestly increases the pro-inflammatory phenotype of naive myelin-treated macrophages. **a** mRNA expression of *Il1b, Nos2, Tnfa, Il6,* and *Ccl5* in bone marrow-derived macrophages (BMDMs, *n* = 6 wells) treated with vehicle, myelin, and the CD36 inhibitor sulfo-N-succinimidyl oleate (SSO, 100 μM) for 24 h. Dotted line represents control cells. **b** mRNA expression of *Il1b, Nos2, Tnfa, Il6,* and *Ccl5* in BMDMs (*n* = 6 wells) treated with vehicle or SSO (100 μM). Data are represented as mean ± s.e.m. *p < 0.05 and **p < 0.01.

**Additional file 3:Supplemental Figure S3.** CD36 inhibition has no effect on experimental autoimmune encephalomyelitis disease progression when treatment starts after disease onset. Disease score of 12-week-old wild-type (wt) mice in which experimental autoimmune encephalomyelitis (EAE) was induced. When a disease score of 0.5 or higher was obtained, animals were injected intraperitoneally with vehicle (*n* = 7 animals) or the CD36 inhibitor sulfo-N-succinimidyl oleate (SSO, 30 mg/kg, *n* = 8 animals) on a daily basis. Data are represented as mean ± s.e.m.

## Data Availability

The datasets used and/or analyzed during the current study are available through the corresponding author on reasonable request.

## Abbreviations

7AAD: 7-Aminoactinomycin D
BMDMs: Bone marrow-derived macrophages
CLP1: Collectin placenta 1
CNS: Central nervous system
DiI: 1,1′-dioctadecyl-3,3,3′,3′-tetramethylindocarbocyanine perchlorate
dMBP: Degenerated myelin basic protein
EAE: Experimental autoimmune encephalomyelitis
HO1: Heme oxygenase 1
LPS: Lipopolysaccharide
LXR: Liver X receptor
MFI: Mean fluorescence intensity
MS: Multiple sclerosis
NO: Nitrite
NRF2: Nuclear factor erythroid 2–related factor 2
NQO1: NAD(P)H:quinone acceptor oxidoreductase 1
ORO: Oil red O
ROS: Reactive oxygen species
SCI: Spinal cord injury
SSO: Sulfo-N-succinimidyl oleate
PPAR: Peroxisome proliferator-activated receptor
