# Supplementary figures and images for: CD36-mediated uptake of myelin debris by macrophages and microglia reduces neuroinflammation
Source: J Neuroinflammation. 2020 Jul 27;17:224. doi: 10.1186/s12974-020-01899-x (PMC7384221; doi:10.1186/s12974-020-01899-x)

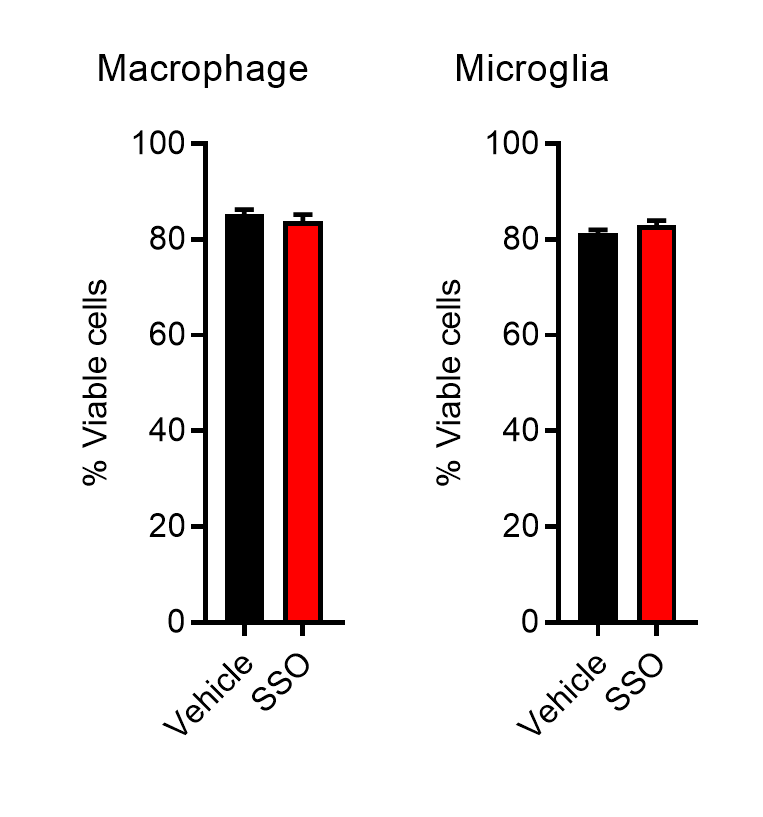

Supplement: Supplementary file 1 — Additional file 1:Supplemental Figure S1. CD36 inhibition does not affect phagocyte viability. Percentage of viable bone marrow-derived macrophages (BMDMs, n = 6 wells) and microglia (n = 4 wells) after treatment with vehicle or the CD36 inhibitor sulfo-N-succinimidyl oleate (SSO, 100 μM) for 24 h. Data are represented as mean ± s.e.m. [file 12974_2020_1899_MOESM1_ESM.png]

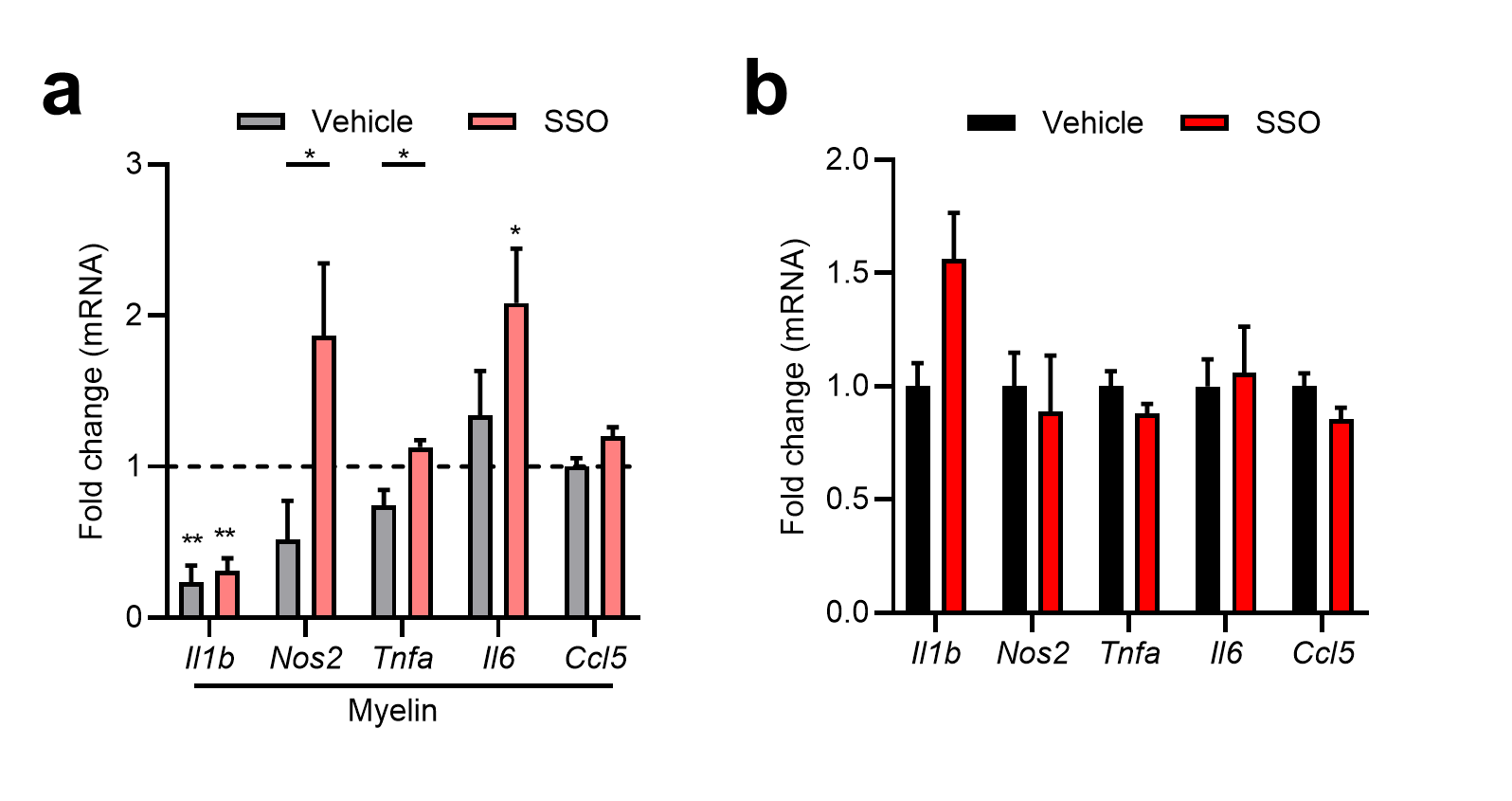

Supplement: Supplementary file 2 — Additional file 2: Supplemental Figure S2. CD36 inhibition modestly increases the pro-inflammatory phenotype of naive myelin-treated macrophages. a mRNA expression of Il1b, Nos2, Tnfa, Il6, and Ccl5 in bone marrow-derived macrophages (BMDMs, n = 6 wells) treated with vehicle, myelin, and the CD36 inhibitor sulfo-N-succinimidyl oleate (SSO, 100 μM) for 24 h. Dotted line represents control cells. b mRNA expression of Il1b, Nos2, Tnfa, Il6, and Ccl5 in BMDMs (n = 6 wells) treated with vehicle or SSO (100 μM). Data are represented as mean ± s.e.m. *p < 0.05 and **p < 0.01. [file 12974_2020_1899_MOESM2_ESM.png]

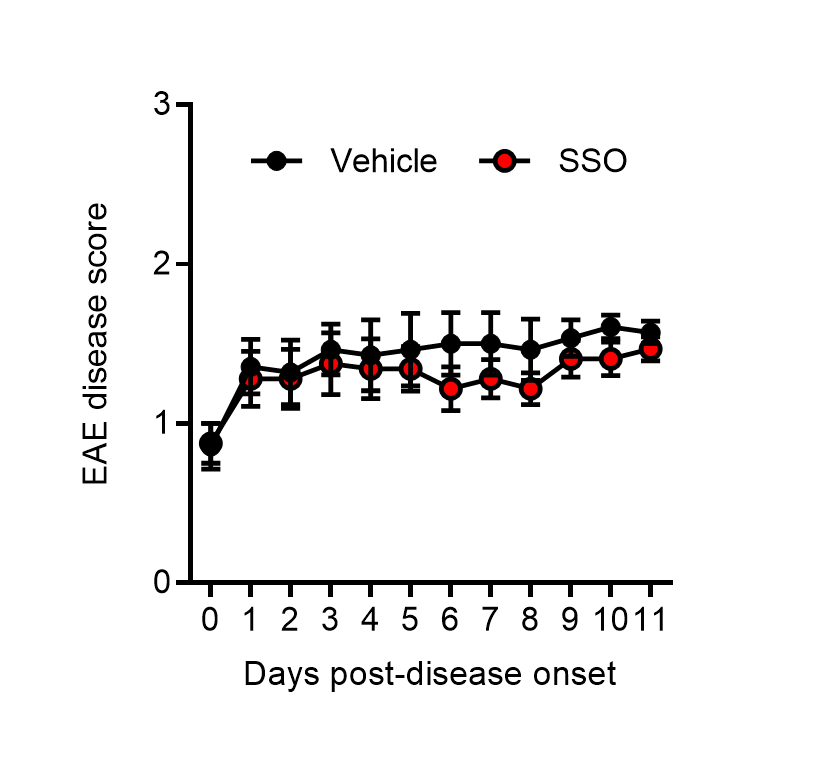

Supplement: Supplementary file 3 — Additional file 3:Supplemental Figure S3. CD36 inhibition has no effect on experimental autoimmune encephalomyelitis disease progression when treatment starts after disease onset. Disease score of 12-week-old wild-type (wt) mice in which experimental autoimmune encephalomyelitis (EAE) was induced. When a disease score of 0.5 or higher was obtained, animals were injected intraperitoneally with vehicle (n = 7 animals) or the CD36 inhibitor sulfo-N-succinimidyl oleate (SSO, 30 mg/kg, n = 8 animals) on a daily basis. Data are represented as mean ± s.e.m. [file 12974_2020_1899_MOESM3_ESM.png]
